# Trap Depth Modulation and Antenna Effect of Organic Ligands for Enhancing Rare‐Earth Long Persistent Luminescence

**DOI:** 10.1002/advs.75829

**Published:** 2026-05-26

**Authors:** Ting Tan, Nian Li, Bangmin Liu, Hao Wang, Xia Huang, Yuanjin Chen, Zhonghao Wang, Chaolong Yang

**Affiliations:** ^1^ School of Materials Science and Engineering Chongqing University of Technology Chongqing China

**Keywords:** energy transfer, long persistent luminescence materials, organic–inorganic hybridization, surface coordination, trap depth modulation

## Abstract

Rare‐earth long persistent luminescence (LPL) materials with unique light‐storage properties show great promise for diverse applications. However, modifying the optical properties of such materials is extremely challenging due to their inherent characteristics. Here, we propose a coordination modification strategy that not only deepens the trap depth but also enhances the light‐capturing capability of rare‐earth LPL materials by introducing organic ligands as an antenna. Unlike previous single‐case demonstrations, this strategy is systematically validated across multiple LPL hosts with different afterglow colors, including green (SrAl_2_O_4_:Eu^2+^, Dy^3+^), red (Sr_0.75_Ca_0.25_S:Eu^2+^), and blue (SrSiO_3_:Eu^2+^, Dy^3+^). Experimental results demonstrate that the afterglow intensity, brightness, and duration of the organic–inorganic hybrid LPL materials have been significantly enhanced compared to the original LPL materials. Based on this performance breakthrough, we further demonstrate the application potential of these materials in diverse scenarios, including flexible displays, intelligent sensors, and multi‐level information encryption. This work not only provides an efficient method for enhancing the performance of multi‐color long persistent luminescence materials but also offers new design insights for developing novel organic–inorganic hybrid luminescent systems.

## Graphical abstract

Different from the current research trends that mainly focus on optimizing a single color system, a universal organic–inorganic hybrid strategy is proposed, aiming to synergistically enhance the luminescent properties of red, green, and blue multi‐color inorganic long‐persistent phosphorescent materials by trap depth modulation and antenna effect of organic ligand.

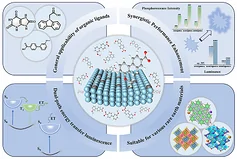

## 1 Introduction

Color, as a fundamental medium for information conveyance, has become deeply integrated into daily life [1]. Its applications range from dynamic anti‐counterfeiting labels [2] that ensure product security to biomedical sensors [3] enabling real‐time health monitoring, bringing tangible convenience and enhanced security to our daily lives. Meanwhile, the precise control and stable output of the three primary colors (red, green, and blue) constitute the foundation for constructing full‐color display systems and advanced optical applications [4, 5, 6]. Long persistent luminescence (LPL) materials are known for their unique light storage properties, which can continue to emit even after removing the excitation source, and have shown significant benefits in many fields, such as bio‐imaging [7, 8, 9], flexible optoelectronics [10, 11, 12], photocatalysis [13, 14, 15], and advanced anti‐counterfeiting [16, 17, 18]. However, although significant progress has been made in optimizing the performance of LPL materials for specific colors, current research strategies remain largely confined to single‐color systems [19, 20, 21, 22]. A general methodology that is universally applicable to red, green, and blue multi‐color systems and capable of synergistically enhancing their performance is still lacking, which greatly hinders the development of full‐color, integrated LPL‐based optoelectronic devices [23, 24, 25]. Therefore, the development of a broad‐spectrum and efficient multicolor LPL enhancement strategy is of great significance for advancing the practical applications of these materials in cutting‐edge fields such as dynamic multicolor displays, high‐security anti‐counterfeiting, and intelligent optical labels.

Afterglow performance, including its intensity and duration, is a key determinant of the application value of rare‐earth LPL materials [26]. However, limited by the inherent properties of inorganic materials, rare‐earth LPL materials face technical bottlenecks such as insufficient luminous intensity and poor light‐capturing capabilities. To overcome these limitations, it is necessary to adopt innovative approaches in material design and processing. Lv et al. synthesized a red‐emitting long‐persistent luminescent (LPL) material, LiYGeO_4_:Mn^2^^+^, via a high‐temperature solid‐state method. The afterglow intensity and duration were significantly enhanced through Bi^3^^+^ co‐doping, which facilitates energy transfer from Bi^3^^+^ to Mn^2^^+^ and optimizes charge trapping processes, demonstrating its potential in information storage and X‐ray detection [27]. In another approach, Yu et al. developed stable red‐emitting CsPbI_3_@zeolite composites through calcination and water treatment. Encapsulation within the zeolite framework effectively removed non‐luminescent phases and reduced shell thickness, leading to a high photoluminescence quantum yield (PLQY) of 31.1% and stability comparable to commercial phosphors [28]. Furthermore, Yang et al. designed LiAlSi_2_O_6_:Sm^3+^ materials, where X‐ray irradiation generated oxygen vacancy defects. This resulted in a notable 57.4% color change under bright‐field illumination and a 93.1% modulation depth of photoluminescence in dark‐field conditions, highlighting its utility in optical modulation [29]. You et al. achieved significant modulation of the emission bandwidth, spanning from ultranarrow to ultrabroad full width at half maximum (FWHM), in MgAlGa_0.7_B_0.3_O_4_:Cr^3+^ by adjusting the Cr^3+^ doping concentration and utilizing energy transfer among the triple emission centers at the Al^3+^, Ga^3+^, and Mg^2+^ sites, thereby enhancing the material's performance [30]. However, the modification process of these strategies is complicated and has poor generalizability. In contrast, recent advances in organic–inorganic hybrid strategies have emerged as a promising platform for high‐performance luminescent materials, including air‐stable organic–inorganic hybrid materials with afterglow durations exceeding 20 h [31] and structurally engineered metal halides achieving near‐unity quantum yield red emission with reversible stimulus‐responsive luminescence through synergistic ligand effects [32]. Therefore, the development of a low‐cost and generalizable strategy to enhance the afterglow properties of materials is important for the development of inorganic LPL materials.

**FIGURE 1:**
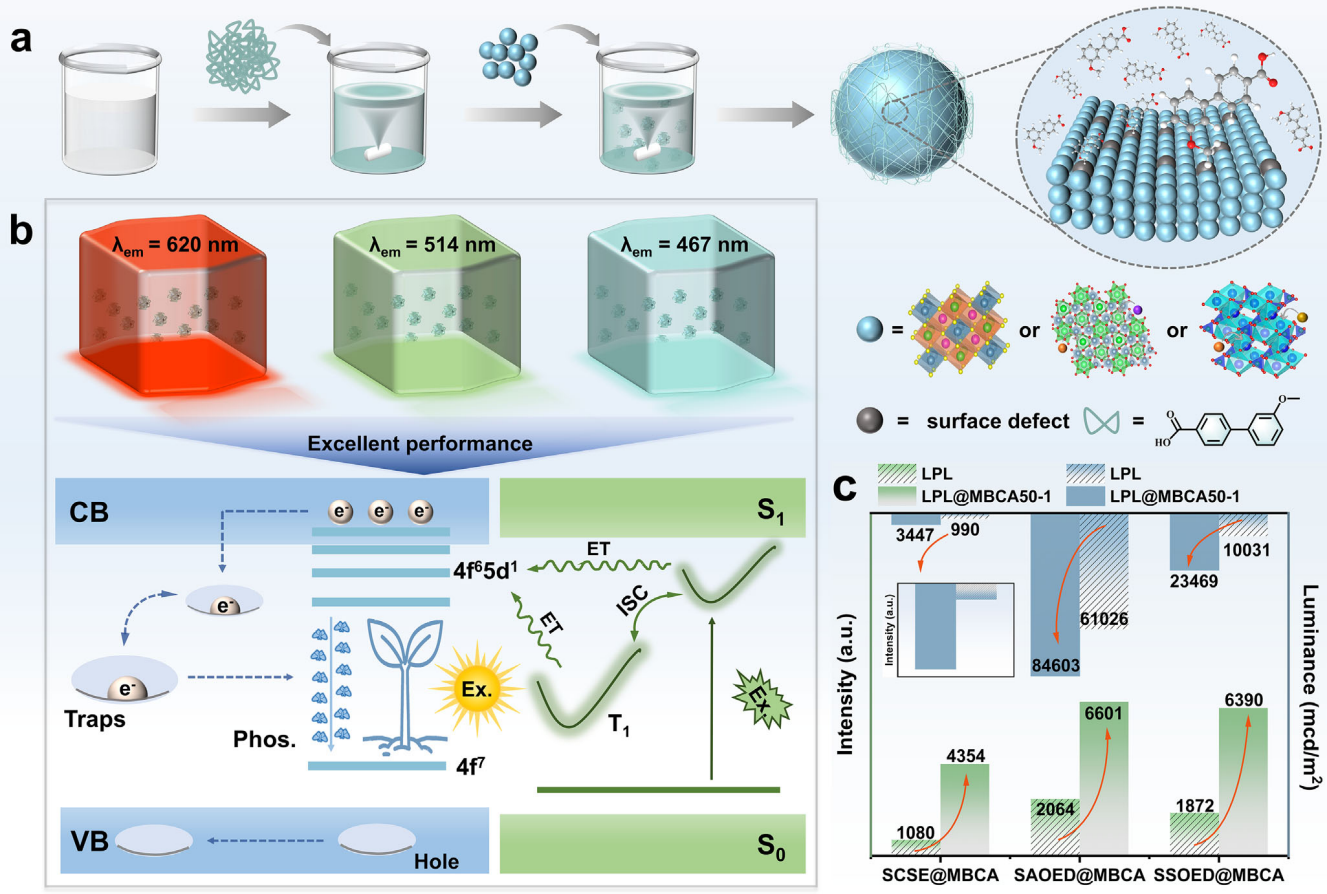
Schematic diagram of the OILPL materials system. (a) Schematic diagram of the preparation process and coordination of OILPL materials. (b) Schematic diagram of the energy transfer mechanism of OILPL materials. (c) Comparison chart of afterglow intensity and initial brightness of OILPL materials and LPL materials under atmospheric conditions.

Inspired by the energy transfer mechanism in organic room‐temperature phosphorescent materials [33, 34], this work proposes a design strategy for enhancing afterglow properties through surface coordination. Unlike previous single‐case demonstrations, this strategy aims to systematically enhance the afterglow properties of LPL materials across multiple hosts and colors. Specifically, a series of organic–inorganic hybridized LPL (OILPL) materials was developed by coordinatively modifying representative inorganic LPL materials with selected organic ligands. The core of this strategy relies on the formation of stable coordination bonds between the functional groups (e.g., carboxyl, amino) of the organic ligands and the metal ions on the inorganic surface. This effectively passivates defect states, such as surface vacancies and non‐coordinated metal ions, thereby suppressing non‐radiative energy loss and consequently enhancing the material's luminescence efficiency and chemical stability. In addition, organic ligands can effectively enhance the ability of materials to capture excitation energy, subsequently acting as energy transfer mediators to significantly boost the afterglow intensity and duration of LPL materials (Figure 1). Based on these considerations, three organic ligands with distinct functional groups, 3'‐methoxybiphenyl‐4‐carboxylic acid (MBCA, containing a carboxyl group), 9‐carbazoleacetic acid (9CAA, containing a carbazole moiety), and uric acid (UA, containing a purine structure), were selected to systematically investigate the structure‐activity relationships governing the enhancement effect. Carboxyl‐containing ligands are known for their strong coordination affinity to metal ions [35], while nitrogen‐containing heterocyclic ligands offer tunable triplet energy levels and high intersystem crossing efficiencies [36]. Purine‐based ligands provide multiple coordination sites for modulating energy transfer [37]. By systematically investigating different ligand‐host combinations, this approach not only demonstrates universality but also provides design guidelines for optimizing organic–inorganic hybrid LPL systems.

Notably, the luminescent properties of the inorganic LPL materials were significantly enhanced after being modified by organic ligands (Figure 1c). Taking MBCA ligands as an example, the afterglow intensity of OILPL materials formed after coordination can be increased by up to three times, the afterglow brightness by up to 2.48 times, and the duration of the afterglow can be extended to a maximum of 210 min. These superior properties enabled us to successfully fabricate a series of functional devices, including flexible films, 3D‐printed architectures, and optical temperature‐sensing labels, thereby highlighting their significant potential for cutting‐edge applications such as low‐visibility lighting and information encryption.

## 2 Results and Discussion

Through coordination assembly between the organic ligand MBCA and the inorganic LPL materials, three LPL@MBCA hybrids were successfully synthesized, namely SCSE@MBCA, SAOED@MBCA, and SSOED@MBCA (Figure 1a). To optimize the material performance, we first systematically investigated the properties of SCSE@MBCA at different mass ratios to identify the optimal formulation. This optimized ratio was then applied to the preparation of SAOED@MBCA and SSOED@MBCA. To confirm the successful synthesis of the OILPL materials, a series of structural and compositional characterizations was conducted, including Fourier transform infrared (FT‐IR) spectroscopy, X‐ray photoelectron spectroscopy (XPS), X‐ray diffraction (XRD), and scanning electron microscopy (SEM).

**FIGURE 2:**
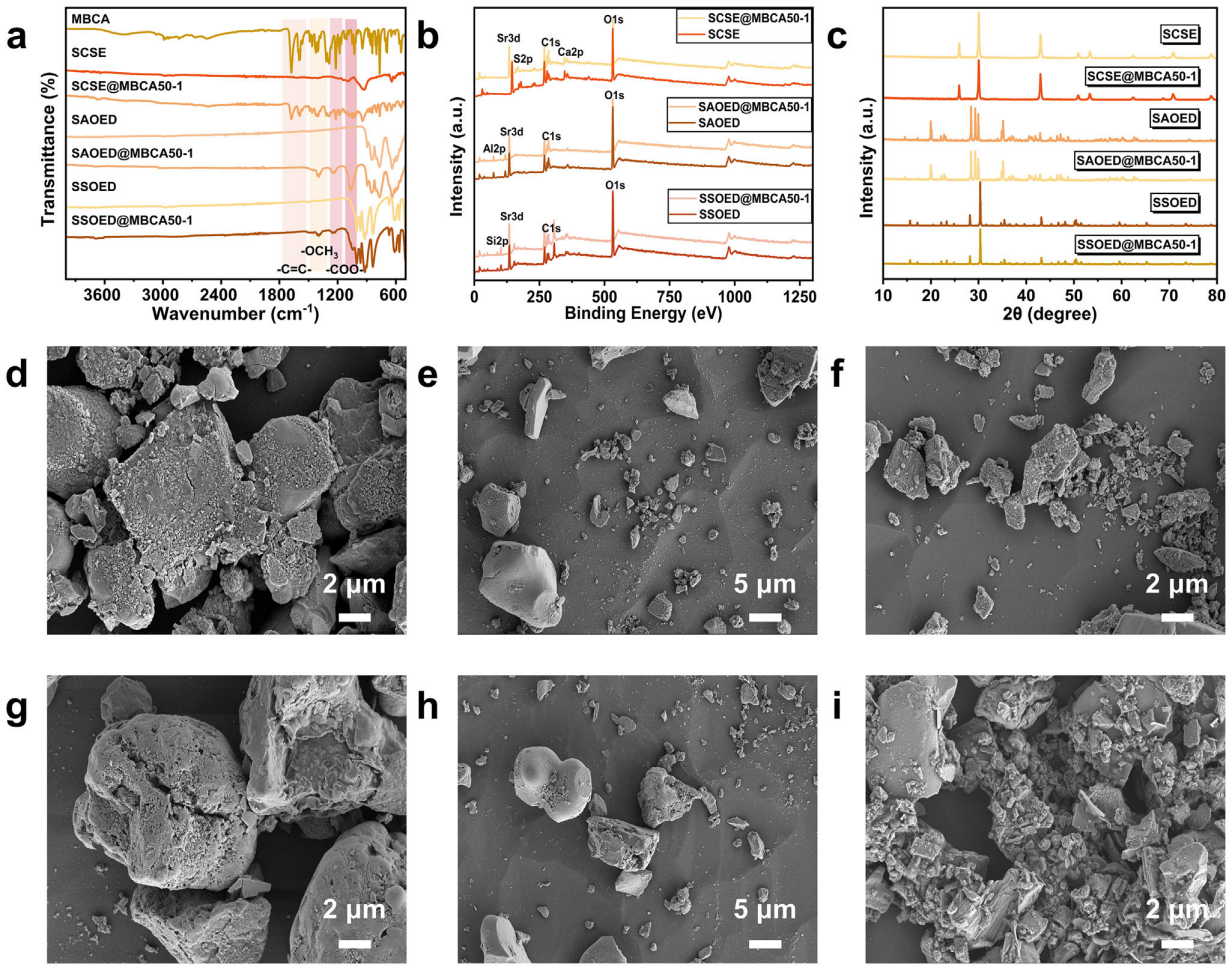
Structure, elemental characterization of OILPL materials. (a) FT‐IR spectra of LPL materials and LPL@MBCA50‐1. (b) XPS spectra of LPL materials, LPL@MBCA50‐1. (c) XRD spectra of LPL materials, LPL@MBCA50‐1. SEM image of (d) SCSE, (e) SAOED, (f) SSOED, (g) SCSE@MBCA50‐1, (h) SAOED@MBCA50‐1, (i) SSOED@MBCA50‐1.

The FT‐IR spectra of all three MBCA‐modified inorganic long‐persistent luminescence materials consistently showed characteristic peaks of organic functional groups, such as benzene ring, carboxyl, and methoxy groups (Figure 2a and Figure S1), confirming the successful coordination of the organic ligand onto the material surfaces. The characteristic peak of the free carboxyl group at 1710 cm^−1^ disappeared in the SCSE@MBCA, with concomitant emergence of two new peaks at 1560 and 1420 cm^−1^ corresponding to the asymmetric and symmetric stretching vibrations of the carboxylate group (‐COO^−^), indicating the formation of ionic bonding between the carboxyl groups and surface Ca^2+^ ions. For SAOED@MBCA, the C═O stretching vibration exhibited a redshift from 1705 cm^−1^, accompanied by the emergence of a characteristic absorption near 1020 cm^−1^ ascribed to an Al─O─C covalent bond, indicating a perturbation of the electronic structure induced by covalent bonding with Al^3+^ ions. For SSOED@MBCAthe appearance of characteristic peaks at 1394 and 1228 cm^−1^, corresponding to coordinated carboxylate groups, along with a new band at 1040 cm^−1^ and the concurrent attenuation of the silanol peak, confirms successful chemical coordination. These data provided compelling evidence for chemical interactions between MBCA and the surface of the inorganic long‐persistent luminescent material. Furthermore, a progressive enhancement in the intensity of the characteristic absorption bands was also observed in the SCSE@MBCA sample series as the MBCA ligand ratio was systematically elevated (Figure S2).

The XPS survey spectra demonstrated that no new characteristic peaks appeared in any of the three LPL materials following MBCA modification, indicating that no additional elemental species were introduced. It is noteworthy that analysis of the high‐resolution XPS spectra revealed modified peak positions (Figure 2b and Figures S3–S8) accompanied by variations in elemental content (Tables S1–S3). Quantitative XPS analysis elucidated distinct bonding mechanisms across the three material systems. For SCSE, the observed elemental variations confirm MBCA modification through coordination, which concurrently facilitates contaminant removal and specific surface functionalization. In the SAOED system, although the overall elemental composition remained largely stable, the marked increase in Al content coupled with the balanced oxygen state indicates ligand anchoring via Al─OOC covalent bonding. For SSOED, the significant reduction in carbon content accompanied by elevated oxygen levels demonstrates successful ligand tethering through silanol condensation. Interestingly, XRD analysis revealed that the organic ligand modification did not induce significant changes in the lattice strain of the inorganic long‐persistent luminescence materials (Figure 2c). These results demonstrate that the organic ligands were successfully coordinated onto the material surfaces without altering their crystal structure.

To further investigate the impact of organic ligand bonding on the microscopic morphology and interfacial structure of the materials, a comparative SEM analysis was conducted on the three types of inorganic long‑persistent luminescent materials and their corresponding organic‐‑inorganic hybrid composites (LPL@MBCA). After modification with MBCA, no significant changes were observed in the particle morphology, size, or dispersion state of any of the samples, indicating that the surface‑modification strategy possesses good precision and controllability without inducing particle agglomeration, regrowth, or macroscopic alteration of the crystal structure (Figure 2d–i). This result is consistent with the surface chemical bonding evidence revealed by FT‑IR and XPS, collectively confirming that the hybridization strategy developed in this work effectively modulates the interfacial states while fully preserving the intrinsic structure of the inorganic matrix, thereby laying an important structural and morphological foundation for the subsequent synergistic enhancement of photophysical properties.

**FIGURE 3:**
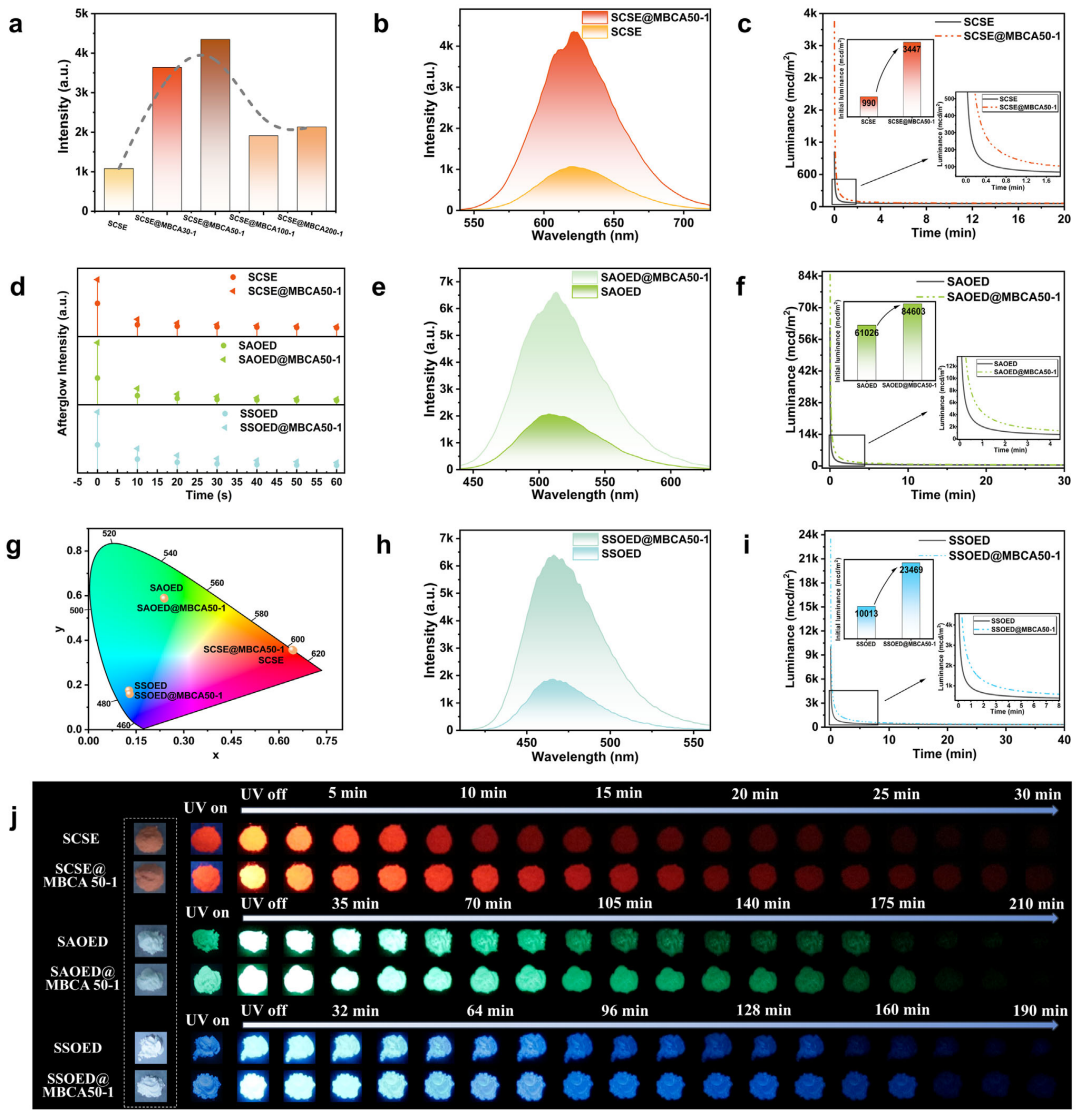
Photophysical properties of OILPL materials. (a) Afterglow intensity of SCSE and SCSE@MBCA at various ratios (λ_ex_ = 365 nm). (b) Afterglow spectrum of SCSE and SCSE@MBCA50‐1 (λ_ex_ = 365 nm). (c) Afterglow brightness decay curves of SCSE and SCSE@MBCA50‐1, with a partially enlarged detail image in the right and an original luminance histogram in the top (λ_ex_ = 365 nm). (d) Afterglow intensity of LPL and LPL@MBCA50‐1 at different times after turning off the excitation source. (e) Afterglow spectrum of SAOED and SAOED@MBCA50‐1. (f) Afterglow brightness decay curves of SAOED and SAOED@MBCA50‐1. (g) CIE coordinate diagram. (h) Afterglow spectrum of SSOED and SSOED@MBCA50‐1. (i) Afterglow brightness decay curves of SSOED and SSOED@MBCA50‐1, with a partially enlarged detail image in the right and an original luminance histogram in the top. (j) Afterglow imaging of LPL and LPL@MBCA50‐1 (λ_ex_ = 365 nm, UV lamp power: 5 W, irradiation time: 60 s).

Subsequently, we conducted a detailed exploration of the optical properties of LPL and LPL@MBCA (Figure 3). To examine the effect of the mass ratio of LPL to MBCA on the afterglow performance, a series of OILPL samples with different ratios were prepared, denoted as SCSE@MBCA30‐1, SCSE@MBCA50‐1, SCSE@MBCA100‐1, and SCSE@MBCA200‐1. After coordination with MBCA, no significant changes in afterglow color or emission peak position were observed, which is confirmed by the high overlap of CIE coordinates and emission peak position before and after modification (Figure 3, Figure S9 and Table S4). Remarkably, it was found that the afterglow intensity of all SCSE@MBCA exceeded that of SCSE. Among them, the SCSE@MBCA50‐1 sample exhibited the most intense afterglow emission (Figure 3a). When the ligand concentration was low, the passivation depth of the trap was insufficient. When the ligand was in excess, it caused competitive absorption of the excitation source and concentration quenching, leading to a decrease in the afterglow properties. The 50–1 sample strikes an optimal balance between these factors, thus exhibiting superior performance and was consequently selected as the representative ratio for further discussion.

Afterglow spectroscopic analysis revealed that the afterglow intensity of all three LPL materials was significantly enhanced upon compositing with MBCA, with approximate increases by factors of 3.0, 2.2, and 2.4 for SCSE, SAOED, and SSOED, respectively (Figure 3b,e,h); corresponding photoluminescence quantum yield (PLQY) measurements further confirmed the luminescence enhancement (Table S5). Compared to the unmodified control, the MBCA‐modified samples showed superior afterglow performance, with increased brightness and extended duration (Figure 3j). The afterglow intensity and brightness decay curves indicate that the OILPL material exhibits significant enhancement in both initial afterglow intensity and brightness (Figure 3c,d,f,i). Specifically, the initial afterglow intensities of SCSE, SAOED, and SSOED increased by approximately 0.62‐, 1.43‐, and 1.37‐fold, respectively (Figure 3d and Table S6), and their initial afterglow brightness increased by approximately 2.48‐, 0.39‐, and 1.34‐fold, respectively. Meanwhile, the intensity decay and brightness attenuation of LPL@MBCA50‐1 were markedly slower than those of pure LPL (Figures S10 and S11), demonstrating that the introduction of MBCA onto the LPL surface significantly enhanced its afterglow performance. Moreover, the modified materials also exhibit favorable photostability and environmental stability (Figures S12 and S13), further underpinning their practical applicability.

Thermoluminescence (TL) is a key technique for analyzing the trap energy levels in LPL materials [38]. Upon linear heating, trapped charge carriers are thermally excited and escape from the traps, thereby emitting light. The TL peak temperature (T_m_) is directly correlated with the trap depth (E). Low‐temperature peaks originate from shallow traps, whose captured carriers can escape readily, contributing to short‐lived but bright initial afterglow. In contrast, high‐temperature peaks correspond to deep traps, from which carriers are released gradually, governing the long‐lasting afterglow duration [39]. Consequently, a higher peak temperature generally indicates a greater trap depth.

**FIGURE 4:**
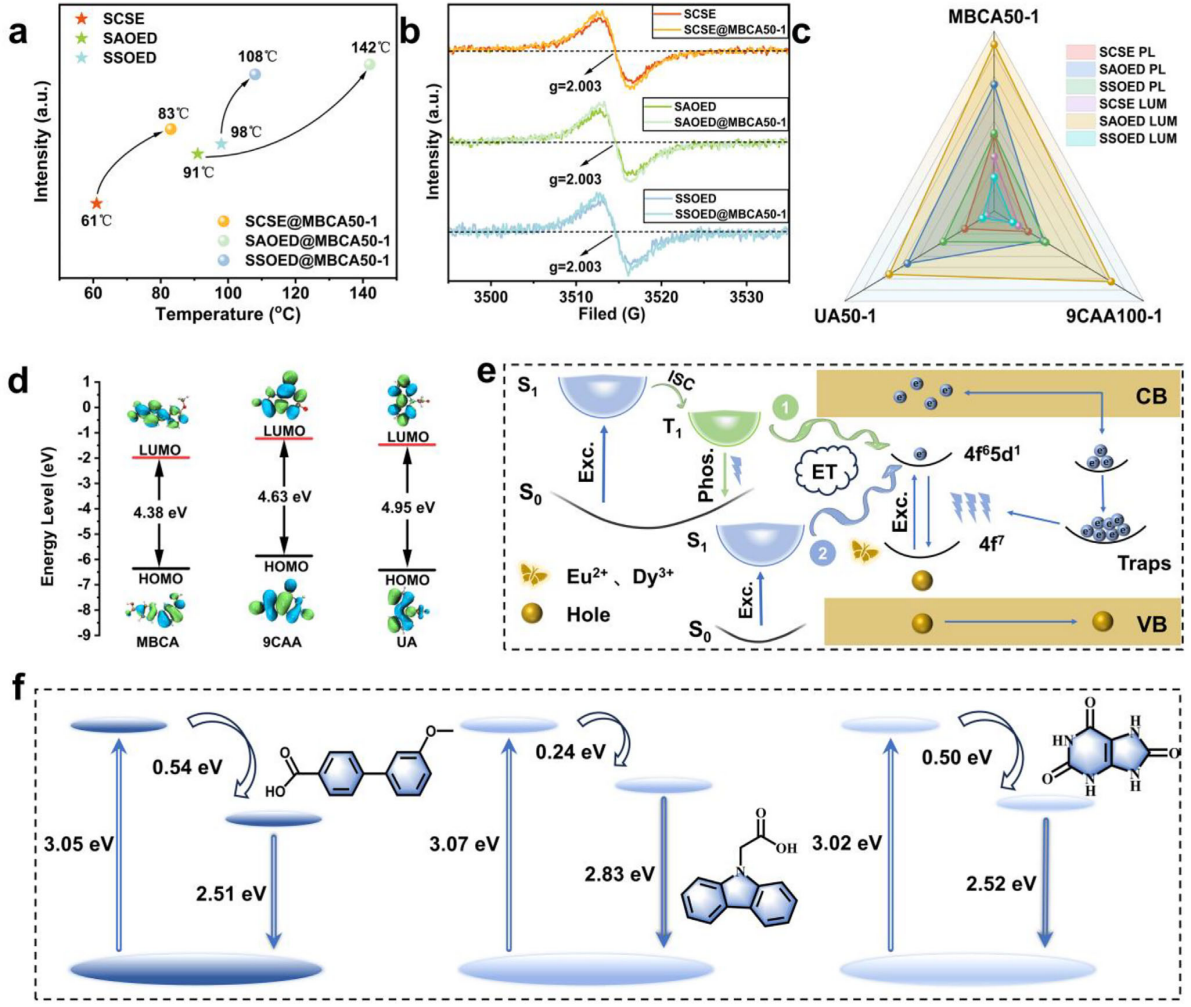
Studies on the luminescence mechanisms and suitability of OILPL materials. (a) Thermoluminescence scatter plot of LPL and LPL@MBCA50‐1. (b) Electron paramagnetic resonance (EPR) spectra of LPL and LPL@MBCA50‐1. (c) Radar plot of afterglow intensity and initial brightness when different ligands are doped into LPL materials. (d) HOMO‐LUMO distribution diagram of MBCA, 9CAA, and UA. (e) Schematic illustration of the RTP emission process in LPL@9CAA and LPL@UA. (f) Energy level diagram for MBCA, 9CAA, UA.

To investigate the trap distribution, TL measurements were performed, which revealed a single broad emission peak for all materials (Figure 4a and Figure S14). It is noteworthy that the OILPL exhibited a systematic shift of their TL peaks toward higher temperatures. The thermoluminescence peak temperatures of SCSE, SAOED, and SSOED increased from 61°C to 83°C, from 91°C to 142°C, and from 98°C to 108°C, respectively. Furthermore, organic ligands and organic–inorganic hybrid materials exhibited excellent thermal stability, ensuring the accuracy of the thermoluminescence curves (Figures S15 and S16). According to the empirical formula E  =  T_m_/500  [40], where E represents the trap depth, and T_m_ is the corresponding peak temperature in Kelvin, the calculated trap depths also increased accordingly. Concretely, the trap depth increased from 0.67 to 0.71 eV for SCSE and from 0.74 to 0.76 eV for SSOED, with SAOED exhibiting the most significant increase from 0.73 to 0.83 eV after the modification. EPR measurements reveal that all samples exhibit distinct paramagnetic resonance signals at g = 2.003, a value typically associated with paramagnetic defects such as oxygen vacancies [41]. Following modification with organic ligands, the EPR signal intensity increases (Figure 4b), while no notable shift in the g‐factor is observed, indicating that the nature of the paramagnetic centers remains unchanged. The signal enhancement is primarily attributed to an increase in surface defect concentration induced by the organic ligands. These additional paramagnetic centers act as effective electron traps, providing direct microscopic evidence for the high‐temperature shift and increased trap depth observed in the thermoluminescence spectra.

Nevertheless, the variation in trap depth is not the sole dominant factor governing the performance optimization. The MBCA molecule exhibits fluorescence emission at room‐temperature, indicating its ability to generate singlet excitons. Furthermore, a distinct phosphorescence peak is observed at 495 nm under low‐temperature conditions (77 K), demonstrating that it is also capable of producing triplet excitons (Figure S17). In the OILPL materials, the MBCA triplet excitons generated upon photoexcitation can transfer energy to the luminescent centers of adjacent LPL materials (Eu^2+^ or Dy^3+^) via energy transfer (ET). XRD patterns confirmed that all composites retained the crystal structure of the pristine inorganic host. Therefore, the observed phenomenon should be attributed to the new energy transfer pathway introduced by MBCA, rather than to structural alterations.

In summary, a synergistic luminescence mechanism is proposed for the LPL@MBCA composites, comprising an early‐stage energy transfer process and a late‐stage trap‐controlled afterglow emission. Distinct differences were observed in the energy transfer pathways to different inorganic LPL materials. For SCSE@MBCA and SAOED@MBCA, energy transfer follows a pathway from the T_1_ energy level of the organic ligand to the inorganic luminescent center. In contrast, the energy transfer in SSOED@MBCA occurs from the S_1_ state to the inorganic luminescent center. These two efficient pathways collectively establish a rapid energy supply channel during the initial afterglow stage, thereby significantly enhancing the initial brightness. Subsequently, the persistent afterglow is predominantly governed by the slow thermal release of charge carriers from material traps. Consequently, the introduction of MBCA not only modifies the trap energy levels and, more importantly, also provides customized early‐stage energy transfer paths for different LPL materials, which synergistically optimizes the overall afterglow performance of the material.

To validate the generality of this strategy, this study further extended the ligands to 9CAA and UA. Initially, the optimal mass ratios for their composites with SCSE were determined through systematic screening for subsequent investigations (Figure S18). In addition to confirming successful ligand coordination, photophysical tests showed that the strategy of using 9CAA or UA effectively enhances the luminescence of three different‐colored LPL materials while preserving their original emission color (Figures S19–S26 and Tables S7 and S8). Notably, the extent of afterglow enhancement varied among the three ligands. Quantitative analysis demonstrated that MBCA provided the most pronounced improvement, followed by 9CAA, while UA exhibited a relatively modest effect (Figure 4c).

To elucidate the structure‐activity relationships underlying the performance differences among the three organic ligands MBCA, 9CAA, and UA, we performed systematic characterization and theoretical analysis. Calculations based on frontier molecular orbital theory reveal that the HOMO‐LUMO energy gaps (ΔE_HL_) for the three ligands are 4.38 eV (MBCA), 4.63 eV (9CAA), and 4.95 eV (UA), respectively (Figure 4d). This increasing trend indicates that MBCA possesses the strongest light‐harvesting capability, which supplies a higher initial exciton concentration for the subsequent energy transfer process and constitutes a critical foundation for its optimal performance.

Further experimental and theoretical analyses were conducted to elucidate the ligand‐dependent energy transfer pathways. As a prerequisite for energy transfer, the phosphorescence and fluorescence spectra of the three ligands were measured (Figure 4f, and Figure S27–S29). Specifically, 9CAA exhibits uniquely high S_1_ and T_1_ energy levels. Its elevated T_1_ level enables a unified energy transfer pathway: singlet excitons are efficiently converted to triplet excitons via intersystem crossing (ISC), after which these long‐lived triplet excitons act as a universal energy relay station, transferring energy to all three inorganic luminescent centers (Pathway 1 in Figure 4e and Figure S30). In contrast, MBCA and UA possess relatively lower T_1_ energy levels, making their transfer pathways more sensitive to the energy level alignment with specific acceptors. For SCSE and SAOED, energy transfer from MBCA and UA proceeds via the triplet‐mediated Pathway 1; however, for SSOED, the lower T_1_ levels of MBCA and UA are energetically mismatched, leading to direct energy transfer from the singlet state (S_1_) to the inorganic luminescent center (Pathway 2 in Figure 4e).

It is important to emphasize that these two pathways do not coexist within a single material. Instead, each hybrid material follows a single, well‐defined energy transfer route determined by the specific combination of ligand and host. Despite these pathway differences at the early energy transfer stage, all three hybrids share a synergistic luminescence mechanism that operates through two consecutive stages (Figure 4e and Figure S30). Efficient early‐stage energy transfer from the organic ligand to the inorganic host (via either T_1_‐mediated or S_1_‐mediated pathways), followed by slow trap release in the later stage, where carriers trapped in the inorganic host's defect states are gradually released, sustaining long‐lived afterglow emission. This synergistic mechanism combines rapid ligand‐host energy transfer with trap regulation, thereby comprehensively enhancing the material's afterglow properties.

**FIGURE 5:**
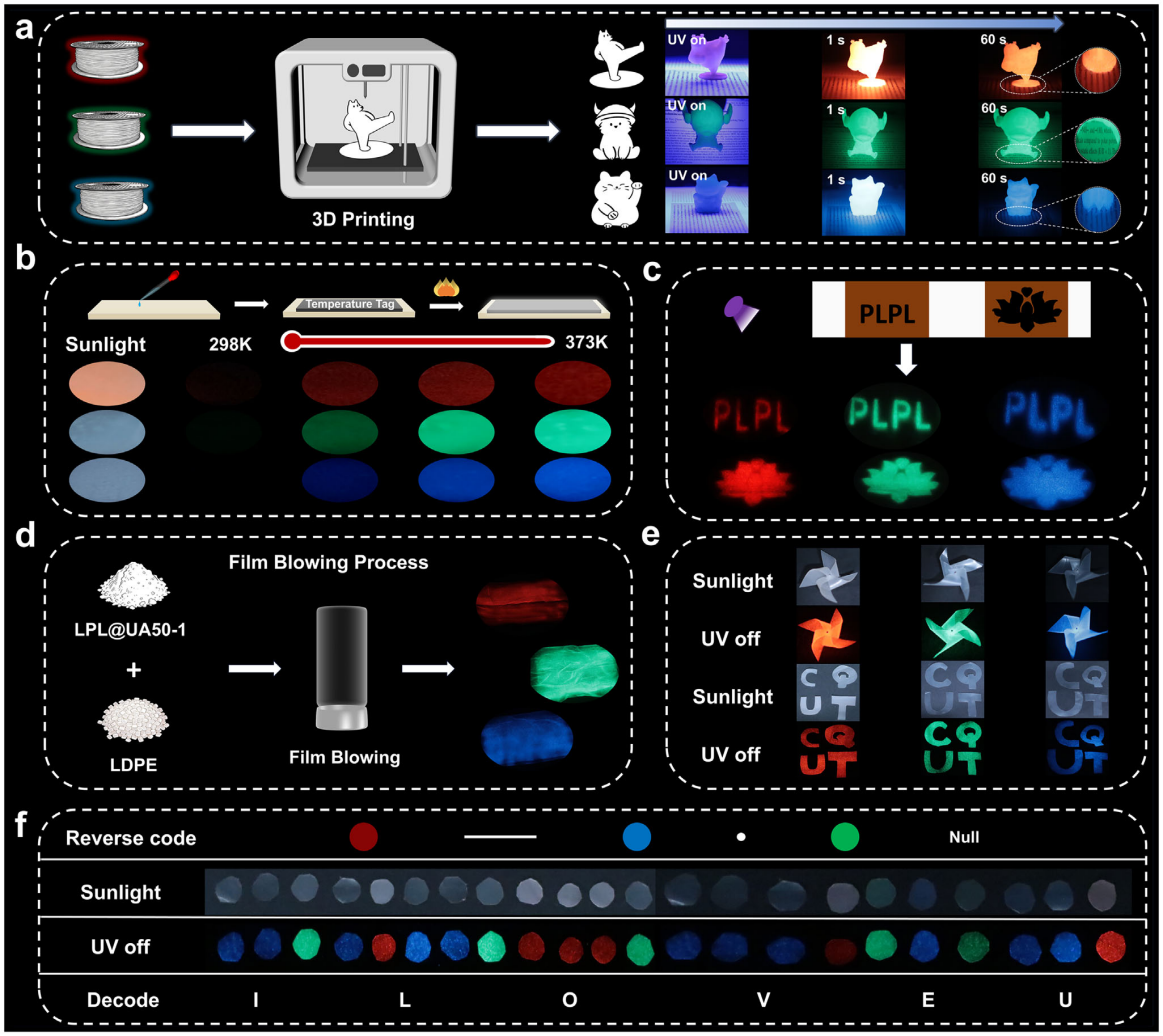
Applications of intrinsic OILPL materials. (a) Physical photos and afterglow photos of the 3D‐printed objects by PLA‐LPL@UA50‐1. (b) Digital photographs about the afterglow emission of EP‐LPL@UA50‐1 composites under different temperatures. (c) Real photograph and afterglow image of the patterns on the LDPE‐LPL@UA50‐1 film. (d) Schematic diagram of the film blowing process. (e) Various transparent, large‐area flexible 3D products were fabricated using LDPE‐LPL@UA50‐1. (f) The encoding and decoding process of Morse code.

Owing to the excellent afterglow properties of the composite material, this study investigates its potential application in 3D printing (Figure S31). By compounding LPL@UA50‐1 with polylactic acid (PLA), a multicolor long‐afterglow 3D printing filament was successfully fabricated. Using the fused deposition modeling (FDM) technique, a finely structured three‐dimensional object was printed with this filament. After excitation under UV light, the printed object exhibited bright trichromatic afterglow emission. Remarkably, the 3D‐printed structure achieves a balance between long‐persistent luminescence duration and high absolute luminance, enabling it to persistently illuminate embedded information in the dark (Figure 5a and Movies S1–S3). This feature demonstrates the potential of such materials for applications in multidimensional information encryption and luminescent displays.

The LPL@UA50‐1 composite material was blended with transparent epoxy resin at a predetermined ratio, coated onto a clean substrate, and cured to form a multicolor long‐persistent optical temperature sensing unit. Based on the thermally stimulated effect, the luminescence intensity of the unit varies sensitively with ambient temperature, enabling visual optical temperature monitoring (Figure 5b). This strategy offers a viable approach for developing novel non‐contact optical temperature sensors.

A flexible film exhibiting multicolor long‐persistent emission was prepared by physically blending low‐density polyethylene (LDPE) with LPL@UA50‐1, followed by melt blending and blow film processing (Figure 5d). The film allows customized patterning such as “PLPL” characters and floral motifs on its surface (Figure 5c). The resulting film demonstrates excellent flexibility and mechanical stability, sustaining repeated bending without delamination or damage (Figures s32–s35), while maintaining stable afterglow and afterglow performance even when folded into various configurations and stretched (Figure 5e and Movie S4). It is noteworthy that the film emits continuous afterglow in the dark that spectrally overlaps with the absorption bands of plant photosynthetic pigments. This property suggests its potential to prolong photosynthesis under natural light conditions, supporting the development of intelligent agricultural covering materials.

Furthermore, leveraging the distinct afterglow colors and lifetimes of different samples, we developed an optical information encryption strategy based on Morse code. In this system, the red afterglow of SCSE@U50‐1 is defined as a dash (“**—**”), the blue afterglow of SSOED@UA50‐1 as a dot (“**·**”), and the green afterglow of SAOED@UA50‐1 as a character spacer. This encryption approach exhibits inherent flexibility, as the encoding rules—for instance, assigning long‐lived afterglow to dots and short‐lived afterglow to dashes—can be dynamically reversed according to predefined logic, thereby enabling the construction of more complex encryption hierarchies. As a proof‐of‐concept, we successfully employed this system to encode and display the extended phrase “I LOVE U” (Figure 5f).

## 3 Conclusions

This work proposes a universal organic–inorganic hybridization strategy for synergistically enhancing the optical physical performance of red, green, and blue multicolor inorganic LPL materials. Our hybridization strategy is distinguished by its broad applicability to various multicolor systems, achieved through coordination between an organic ligand and multiple inorganic LPL hosts (SCSE, SAOED, SSOED), contrasting with the common focus on single‐color optimization. Mechanism analysis indicates that the organic ligand acts as an effective energy capture and transfer unit, and its energy transfer process works synergistically with the intrinsic trap effects of the materials, collectively promoting comprehensive afterglow enhancement. Driven by this synergistic mechanism, all tested materials demonstrated significantly enhanced performance. The afterglow intensity of SCSE, SAOED, and SSOED was enhanced by approximately 3.0, 2.2, and 2.4 times, respectively. The initial brightness was increased by about 2.48, 0.39, and 1.34 times, respectively. The afterglow duration was extended to 30, 210, and 190 min, respectively. Utilizing the enhanced materials, we demonstrated the strategy's application potential across various forms, including afterglow films, 3D‐printed structures, and optical temperature‐sensing labels. These implementations confirm the material's feasibility in low‐light illumination, flexible displays, high‐temperature real‐time monitoring, and information encryption. In summary, this strategy provides a generic approach for constructing multicolor long‐persistent luminescence materials and offers a design methodology for advancing next‐generation LPL systems.

## 4 Experimental Section

### 4.1 Preparation of SCSE@MBCA Solid Powder

The preparation method for SCSE@MBCA was outlined as follows: MBCA (0.02、0.012、0.006、0.003 g) was placed in a beaker, and 60 mL DMF was added. The mixture was sonicated for 5 min to ensure complete dissolution of MBCA in DMF. Subsequently, Sr_0.75_Ca_0.25_S:Eu^2+^ (0.6 g) was added, heated, and stirred (temperature: 90°C) for 9 h. The product was then filtered and placed in an oven at 80°C for 12 h, and dried thoroughly to obtain solid powder SCSE@MBCA (30:1, 50:1, 100:1, and 200:1).

### 4.2 Preparation of SAOED@MBCA Solid Powder

The preparation method for SCSE@MBCA was outlined as follows: MBCA (0.012 g) was placed in a beaker, and 60 mL DMF was added. The mixture was sonicated for 5 min to ensure complete dissolution of MBCA in DMF. Subsequently, SrAl_2_O_4_:Eu^2+^, Dy^3+^ (0.6 g) was added, heated, and stirred (temperature: 90°C) for 9 h. The product was then filtered and placed in an oven at 80°C for 12 h, and dried thoroughly to obtain solid powder SAOED@MBCA 50:1

### 4.3 Preparation of SSOED@MBCA Solid Powder

The preparation method for SCSE@MBCA was outlined as follows: MBCA (0.012 g) was placed in a beaker, and 60 mL DMF was added. The mixture was sonicated for 5 min to ensure complete dissolution of MBCA in DMF. Subsequently, SrSiO_3_:Eu^2+^, Dy^3+^ (0.6 g) was added, heated, and stirred (temperature: 90°C) for 9 h. The product was then filtered and placed in an oven at 80°C for 12 h, and dried thoroughly to obtain solid powder SSOED@MBCA 50:1.

### 4.4 Preparation of LDPE–LPL@UA50‐1 Film

First, 100 g of low‐density polyethylene (LDPE) was accurately weighed and thoroughly physically blended with 2 g of the LPL@UA composite material (achieving a mass ratio of 50:1). The mixture was then melt‐extruded and pelletized using a twin‐screw extruder. Subsequently, the resulting pellets were processed into film via a blow molding technique, ultimately yielding three types of phosphorescent films capable of emitting red, green, and blue afterglow, respectively. The LDPE used in this study was commercial grade 2426H (Sinopec, China), with a molecular weight (M_w_) in the range of 50 000–500 000 g/mol, a melt flow index (MFI) of approximately 1.9 g/10 min (190°C/2.16 kg), and a density of 0.924 g/cm^3^.

### 4.5 Preparation of PLA‐LPL@UA50‐1 3D‐Printed Objects

First, 5 kg of polylactic acid (PLA) was thoroughly dry‐mixed with 100 g of a specific LPL@UA composite material (corresponding to a mass ratio of PLA to LPL@UA of 50:1). Subsequently, the mixture was processed via a melt‐blending process using a twin‐screw extruder, ultimately fabricating three types of 3D printing filaments exhibiting three distinct colors. Based on a predefined three‐dimensional model, the corresponding three‐dimensional structural components were successfully fabricated using the aforementioned filaments on a fused deposition modeling (FDM) 3D printer. The PLA used was commercial grade 4032D (NatureWorks, USA), with a density of 1.24 g/cm^3^, a melt flow index of 7 g/10 min (210°C, 2.16 kg), a melting point (T_m_) of approximately 162°C (range: 155°C–170°C), an average molecular weight (M_w_) of ∼155 000 g/mol, and a D‐isomer content of ∼1.5%.

## Author Contributions

**Ting Tan**: methodology, conceptualization, data curation, formal analysis, validation, investigation, visualization, writing – original draft, writing – review and editing, software. **Hao Wang**: conceptualization, methodology, software, data curation, formal analysis, validation, investigation, visualization, writing – review and editing. **Xia Huang**: methodology, software, data curation, formal analysis, validation, investigation, visualization, writing – review and editing. **Bangmin Liu**: conceptualization, methodology, software, data curation, formal analysis, writing – review and editing, investigation, validation, visualization. **Nian Li**: methodology, software, investigation, formal analysis, writing – review and editing, data curation, conceptualization, validation, visualization. **Chaolong Yang**: conceptualization, investigation, funding acquisition, writing – review and editing, visualization, validation, methodology, software, formal analysis, project administration, resources, supervision, data curation. **Zhonghao Wang**: conceptualization, methodology, writing – review and editing, visualization, validation, investigation, data curation, resources, project administration, formal analysis, software, supervision. **Yuanjin Chen**: methodology, software, data curation, formal analysis, investigation, validation, writing – review and editing, visualization.

## Conflicts of Interest

The authors declare no conflicts of interest.

## Supporting information




**Supporting File 1**: advs75829‐sup‐0001‐SuppMat.docx.

**Supporting File 2**: advs75829‐sup‐0002‐MovieS1.mp4.

**Supporting File 3**: advs75829‐sup‐0003‐MovieS2.mp4.

**Supporting File 4**: advs75829‐sup‐0004‐MovieS3.mp4.

**Supporting File 5**: advs75829‐sup‐0005‐MovieS4.mp4.

## Data Availability

The data that support the findings of this study are available in the supplementary material of this article.
