# Supplementary material for: Trap Depth Modulation and Antenna Effect of Organic Ligands for Enhancing Rare‐Earth Long Persistent Luminescence
Source: Adv Sci (Weinh). 2026 May 26;13(46):e75829. doi: 10.1002/advs.75829 (PMC13335827; doi:10.1002/advs.75829)
Supplement: Supplementary file 1 [file ADVS-13-e75829-s005.docx]

Supporting Information

**Trap Depth Modulation and Antenna Effect of Organic Ligands for Enhancing Rare-Earth Long Persistent Luminescence**

Ting Tan, Nian Li, Bangmin Liu, Hao Wang, Xia Huang, Yuanjin Chen, Zhonghao Wang*, and Chaolong Yang*

**Table of Counts**

**EXPERIMENTAL PROCEDURES**

**I.REAGENTS AND MATERIALS**

**II.CHARACTERIZATION**

**Ⅲ.THEORETICAL CALCULATION METHOD**

**IV. SUPPLEMENTARY FIGURES AND TABLES**

**V.SUPPORTING MOVIES**

**Experimental Procedures**

**I. Reagents and materials**

The following reagents used in experiments, including 3'-Methoxy-4-biphenylcarboxylic acid (MBCA), were purchased from bidepharmatech, 9-Carbazoleacetic acid (9CAA) were purchased from aladdin-e and Uric acid (UA) were purchased from leyan. *N, N*-Dimethylformamide (DMF, Analytical Reagent) was purchased from KeLong chemical. Sr_0.75_Ca_0.25_S: Eu^2+^ (SCSE), SrAl₂O₄:Eu²⁺, Dy³⁺(SAOED), SrSiO₃:Eu²⁺, Dy³⁺(SSOED) was purchased from LuMing Technology Group Limited. Unless otherwise stated, all the chemicals were used directly without further purification.

**Structural formula of organic ligands**

3'-Methoxy-4-biphenylcarboxylic acid (MBCA)

9-Carbazoleacetic acid (9CAA)

Uric acid (UA)

**Ⅱ.Characterization**

Powder X-ray diffraction (XRD) measurements were recorded on a Japan Rigaku SmartLab SE using Cu Kα radiation with a 2θ range of 5-80°, 40 KeV, and 50 mA at a scanning rate of 10 min^-1^ (2θ) at room temperature. X-ray photoelectron spectroscopy (XPS) was performed on a America Thermo ESCALAB 250X. FTIR spectra were carried out using a PerkinElmer Fourier transform infrared spectrometer. Scanning electron microscopy (SEM) images were obtained with a JCM-7000.

Phosphorescence spectra were measured at both room temperature and 77 K on a Hitachi F-4700 fluorescence spectrophotometer. Afterglow intensity decay curves at room temperature were measured on an F97pro fluorescence spectrophotometer. This test was carried out after closing the light gate and manually irradiating with a UV lamp and removing the lamp (afterglow intensity decay curves: excitation wavelength: 365 nm, power: 5 W, excitation time: 30 s). The afterglow luminance decay curve at room temperature was measured by a PR-305 detector (excitation time: 40 s; steady state interval: 0.1 s). Thermoluminescent spectra were measured on an FJ-427A1 Microcomputer thermoluminescent dosimeter (temperature range: 30-300 ^o^C, temperature rise rate: 1 ^o^C s^-1^). The LDPE-LPL@MBCA50-1 film was fabricated using an RM-200A torque rheometer and FB-300 blown film extruder. The PLA-LPL@MBCA50-1 3D printing filament is manufactured using the SHSJ45 extruder. This 3D-printed object was manufactured using the HORI Z300+ Printer. The tensile test was carried out using the SUST microcomputer-controlled electronic universal testing machine. Photographs and videos were captured with a Canon EOS 80D camera, and detailed shooting parameters can be found in Table S9.

Throughout this paper, the term LPL@MBCA refers to the class of organic-inorganic hybrid long persistent luminescent materials obtained by modifying inorganic LPL hosts with the organic ligand MBCA, such as SCSE@MBCA, where the host material is indicated before the "@" symbol and the organic ligand after it.

**Ⅲ.Theoretical calculation method**

The ground-state geometry was optimized using the ORCA 5.0.4 software package at the B3LYP/6-311G* level. The HOMO and LUMO orbitals were plotted using Multiwfn and VMD program.

**Ⅳ.Supplementary figures and tables**

**Figure S1.** FT-TR spectra of LPL and LPL@MBCA50-1.

**Figure S2.** FT-TR spectra of SCSE and SCSE@MBCA at different ratios.

**Figure S3.** XPS high-resolution a) C 1s, b) O 1s, c) Ca 2p, d) S 2p, e) Sr 3d, and f) Eu 3d spectra of SCSE.

**Figure S4.** XPS high-resolution a) C 1s, b) O 1s, c) Ca 2p, d) S 2p, e) Sr 3d, and f) Eu 3d spectra of SCSE@MBCA50-1.

**Figure S5.** XPS high-resolution a) C 1s, b) O 1s, c) Al 2p, d) Sr 3d, and e) Eu 3d spectra of SAOED.

**Figure S6.** XPS high-resolution a) C 1s, b) O 1s, c) Al 2p, d) Sr 3d, and e) Eu 3d spectra of SAOED@MBCA50-1.

**Figure S7.** XPS high-resolution a) C 1s, b) O 1s, c) Si 2p, d) Sr 3d, and e) Eu 3d spectra of SSOED.

**Figure S8.** XPS high-resolution a) C 1s, b) O 1s, c) Si 2p, d) Sr 3d, and e) Eu 3d spectra of SSOED@MBCA50-1.

**Figure S9.** CIE coordinate diagrams of SCSE, SCSE@MBCA30-1, SCSE@MBCA50-1, SCSE@MBCA100-1, SCSE@MBCA200-1.

**Figure S10.** The afterglow brightness decay curves of LPL and LPL@MBCA50-1 (excitation wavelength λ_ex_ = 365 nm).

**Figure S11.** The afterglow decay curves of LPL and LPL@MBCA50-1 series materials.

**Figure S12.** Photostability test of LPL@MBCA50-1 under 365 nm UV irradiation.

**Figure S13.** Afterglow spectra of LPL@MBCA50-1 stored in ambient conditions for different periods of time.

**Figure S14.** Thermoluminescence spectrum of LPL and LPL@MBCA50-1.

**Figure S15.** The TGA curves of MBCA, 9CAA, and UA.

**Figure S16.** The afterglow intensity of the LPL@MBCA50-1 during heating-cooling cycles.

**Figure S17.** a) Phosphorescence spectrum of MBCA at 77 K; b) Fluorescence spectrum of MBCA at room temperature.

**Figure S18.** Afterglow spectrum of SCSE and a)SCSE@9CAA and b)SCSE@UA at various ratios. The figure shows the spectrum from the first scan (λ_ex_ = 365 nm).

**Figure S19.** FT-IR spectra of a) LPL and LPL@9CAA100-1, b) LPL and LPL@UA50-1.

**Figure S20. Bar chart of the phosphorescence intensity enhancement for a) LPL@9CAA100-1 and b) LPL@UA50-1.**

**Figure S21.** Afterglow brightness decay curves of LPL and LPL@9CAA100-1 (λ_ex_ = 365 nm), with a partially enlarged detail image in the right and an original luminance histogram in the top.

**Figure S22.** Afterglow brightness decay curves of LPL and LPL@UA50-1, with a partially enlarged detail image on the right and an original luminance histogram at the top.

**Figure S23.** Afterglow imaging of LPL and LPL@9CAA100-1 (λ_ex_ = 365 nm, UV lamp power: 5 W, irradiation time: 60 s).

**Figure S24.**Afterglow imaging of LPL and LPL@UA50-1 (λ_ex_ = 365 nm, UV lamp power: 5 W, irradiation time: 60 s).

**Figure S25.** CIE coordinate diagram corresponding to a) LPL@9CAA100-1 and b) LPL@UA50-1.

**Figure S26.** Thermoluminescence spectrum of a) LPL and LPL@9CAA100-1, and b) LPL and LPL@UA100-1.

**Figure S27.** Phosphorescence spectrum of a) 9CAA and b) UA at 77 K.

**Figure S28.** Fluorescence spectrum of a) MBCA, b) 9CAA, and b) UA at room temperature (λ_ex_ = 365 nm).

**Figure S29.** Photoluminescence spectrum of a) SCSE, b) SSOED, and b) SAOED at room temperature (λ_ex_ = 365 nm).

**Figure S30.** Energy transfer pathways of different ligands and different LPL mate@rials.

**Figure S31.** Three-color long-lasting glow 3D printing filament physical image and afterglow photo.

**Figure S32.** The stress-strain curve of LDPE-LPL@UA50-1 film.

**Figure S33.** The emission images of the folded LDPE-LPL@UA50-1 film (λ_ex_ = 365 nm, Power = 5 W, the irradiation time is 5 s).

**Figure S34.** The real photo and afterglow photo of LDPE-LPL@UA50-1 film when pulling the reactor (λ_ex_ = 365 nm, Power = 5 W, the irradiation time is 5 s).

**Figure S35.** Physical photos of LDPE-LPL@UA50-1 film under different degrees of stretching.

**Table S1**. Percentage content of each element in SCSE and SCSE@MBCA50-1.

| **SCSE** | | **SCSE@MBCA 50-1** | |
| --- | --- | --- | --- |
| **Element** | **At%** | **Element** | **At%** |
| C | 34.40 | C | 11.94 |
| O | 27.52 | O | 38.93 |
| S | 9.29 | S | 12.81 |
| Sr | 7.06 | Sr | 8.65 |
| Ca | 1.03 | Ca | 2.88 |
| Eu | 0.02 | Eu | 0.02 |

**Table S2.** Percentage content of each element in SAOED and SAOED@MBCA50-1.

| **SAOED** | | **SAOED@MBCA 50-1** | |
| --- | --- | --- | --- |
| **Element** | **At%** | **Element** | **At%** |
| C | 28.29 | C | 27.97 |
| O | 35.70 | O | 34.98 |
| Al | 9.19 | Al | 10.50 |
| Sr | 4.32 | Sr | 4.29 |
| Eu | 0.02 | Eu | 0.01 |

**Table S3.** Percentage content of each element in SSOED and SSOED@MBCA50-1.

| **SSOED** | | **SSOED@MBCA50-1** | |
| --- | --- | --- | --- |
| **Element** | **At%** | **Element** | **At%** |
| C | 28.13 | C | 7.10 |
| O | 35.22 | O | 48.26 |
| Si | 9.82 | Si | 9.13 |
| Sr | 5.99 | Sr | 5.87 |
| Eu | 0.02 | Eu | 0.01 |

**Table S4.** The afterglow emission peak positions of LPL and LPL@MBCA materials.

| **Sample** | **Emission Peak (nm)** | **Sample** | **Emission Peak (nm)** |
| --- | --- | --- | --- |
| SCSE | 620.4 | SCSE@MBCA | 620.6 |
| SAOED | 514 | SAOED@MBCA | 514.2 |
| SSOED | 470 | SSOED@MBCA | 468.4 |

**Table S5.** The photoluminescence quantum yield of LPL and LPL@MBCA50-1 at room temperature.

| **Sample** | **PLQY (%)** |
| --- | --- |
| SCSE | 57.42 |
| SCSE@MBCA | 77.94 |
| SAOED | 21.23 |
| SAOED@MBCA | 68.10 |
| SSOED | 17.11 |
| SSOED@MBCA | 43.39 |

**Table S6.** Initial afterglow intensity in LPL and LPL@MBCA50-1.

| **LPL** | | **LPL@MBCA50-1** | |
| --- | --- | --- | --- |
| **Sample** | **Intensity (a.u.)** | **Sample** | **Intensity (a.u.)** |
| SCSE | 668 | SCSE@MBCA50-1 | 1085 |
| SAOED | 3586 | SAOED@MBCA50-1 | 8715 |
| SSOED | 2806 | SSOED@MBCA50-1 | 6653 |

**Table S7.** The afterglow emission peak positions of LPL and LPL@9CAA materials.

| **Sample** | **Emission Peak (nm)** | **Sample** | **Emission Peak (nm)** |
| --- | --- | --- | --- |
| SCSE | 620.4 | SCSE@9CAA | 620.8 |
| SAOED | 514 | SAOED@9CAA | 514.4 |
| SSOED | 470 | SSOED@9CAA | 468.6 |

**Table S8.** The afterglow emission peak positions of LPL and LPL@UA materials.

| **Sample** | **Emission Peak (nm)** | **Sample** | **Emission Peak (nm)** |
| --- | --- | --- | --- |
| SCSE | 620.4 | SCSE@UA | 620.5 |
| SAOED | 514 | SAOED@UA | 514.2 |
| SSOED | 470 | SSOED@UA | 469 |

**Table S9.** Shooting parameters for afterglow photographs presented in this work.

| **Sample Photographs** | **Exposure Time** | **ISO Sensitivity** | **Aperture** | **Ambient Light Conditions** |
| --- | --- | --- | --- | --- |
| Movies | 1/30 s | 10000 | f/2 | under dark conditions |
| 3D printing filament | 1/25 s | 8000 | f/2 |  |
| 3D printed objects | 1/25 s | 10000 | f/2 |  |
| film | 1/4 s | 12800 | f/2 |  |

**Ⅴ. Supporting movies**

**Movie S1:** The afterglow movie of the 3D printed object made by PLA@SCSE@UA50-1. The excitation wavelength is 365 nanometers, the power of the ultraviolet lamp is 5 watts, and the exposure time is 10 seconds. This movie is played at 5 times the normal speed.

**Movie S2:** The afterglow movie of the 3D printed object made by PLA@SAOED@UA50-1. The excitation wavelength is 365 nanometers, the power of the ultraviolet lamp is 5 watts, and the exposure time is 10 seconds. This movie is played at 5 times the normal speed.

**Movie S3:** The afterglow movie of the 3D printed object made by PLA@SSOED@UA50-1. The excitation wavelength is 365 nanometers, the power of the ultraviolet lamp is 5 watts, and the exposure time is 10 seconds. This movie is played at 5 times the normal speed.

**Movie S4:** Afterglow movie of LDPE-LPL@UA50-1 film. The sequence of the samples is LDPE-SCSE@UA50-1, LDPE-SSOED@UA50-1, LDPE-SAOED@UA50-1 in turn. The excitation wavelength is 365 nm, the power of UV lamp is 5 W, irradiation time is 5 s. The movie is played at three times the speed.
